# 27-hydroxycholesterol linked high cholesterol diet to lung adenocarcinoma metastasis

**DOI:** 10.1038/s41388-022-02285-y

**Published:** 2022-04-04

**Authors:** Xingkai Li, Hengchi Chen, Lizhen Zhang, Li Chen, Wei Wei, Shugeng Gao, Qi Xue, Yue Li, Bing Wang, Jiagen Li, Yushun Gao, Yanliang Lin

**Affiliations:** 1 Department of Thoracic Surgery, National Cancer Center/National Clinical Research Center for Cancer/Cancer Hospital, Chinese Academy of Medical Sciences and Peking Union Medical College, Beijing, China; 2 Department of Central Laboratory, Shandong Provincial Hospital Affiliated to Shandong First Medical University, Jinan, China; 3 Department of Central Laboratory, Shandong Provincial Hospital Affiliated to Shandong University, Jinan, China; 4 Shandong provincial Eco-environment Monitoring Center, Jinan, China; 5 Department of Anesthesiology, Peking University Third Hospital, Beijing, China

**Keywords:** Cancer metabolism, Non-small-cell lung cancer

## Abstract

Dietary cholesterol has been implicated to promote lung cancer. Lung adenocarcinoma (LAC) is a main type of lung cancer, whereas the functional mechanism of cholesterol in LAC remained largely unknown. In the present study, we evidenced that cholesterol promoted cell proliferation and invasion of LAC in vitro as well as LAC metastasis in vivo. Cyp27A1 knockdown reduced the cholesterol-induced LAC cells proliferation and invasion. In contrast, Cyp7B1 knockdown enhanced the effect of cholesterol on LAC cells proliferation and invasion. Furthermore, Cyp27A1 deficiency remarkably reduced high cholesterol-induced LAC metastasis in vivo. Mechanism investigation demonstrated that exposure of LAC cells to 27-hydroxycholesterol induced the phosphorylation of AKT and NFκB p65, and promoted the expression of peptidylprolyl isomerase B (PPIB), especially in the coculture with THP1-derived macrophage. Meanwhile, 27-hydroxycholesterol induced the secretion of FGF2 and IL-6, which contributed to the expression of snail and vimentin. Luciferase report assay and ChIP assay confirmed that NFκB p65 controlled the transcription of PPIB. Inhibiting NFκB p65 activation reduced PPIB expression. PPIB inhibition reduced 27-hydroxycholesterol-induced expression of snail and vimentin. These results indicated that 27-hydroxycholesterol linked high cholesterol and LAC metastasis by regulating NFκB/PPIB axis and the secretion of FGF2 and IL-6.

## Introduction

High fat diet and obesity have been closely associated with cancer development [1]. Elevating dietary fat intake increases prostate cancer risk [1]. Total cholesterol is significantly correlated to overall survival in non-small-cell lung cancer patients with epidermal growth factor receptor mutations [2]. Dietary cholesterol has been demonstrated to increase risk of lung cancer [3, 4]. Furthermore, cholesterol reduces the sensitivity of platinum-based chemotherapy in lung adenocarcinoma (LAC) [5]. Fluvastatin, an inhibitor of 3-hydroxy-3-methylglutaryl coenzyme A (HMG-CoA) reductase that is a key enzyme of cholesterol synthesis, suppresses bone metastasis of LAC [6]. Thus, cholesterol plays an important role in the progression of LAC, but the mechanism of which remains largely unknown.

Tumor metastasis is the main cause of high mortality and poor prognosis of LAC [7]. However, the function of cholesterol metabolism in LAC metastasis is still mysterious. Our previous findings have evidenced that 4-cholesten-3-one, an oxidative product of cholesterol, prevents LAC metastasis by regulating translocation of HMGB1, HIF1α and caveolin-1 [8], but 25-hydroxycholesterol promotes the migration and invasion of LAC cells at LXR-dependent and independent manners [9]. The contradictory functions of cholesterol metabolites likely lead to the indeterminate relation between high cholesterol and poor prognosis of LAC. It is necessary to exploit the vital factors for clarifying the role of cholesterol in LAC proliferation and metastasis.

27-hydroxycholesterol is the most prevalent circulating oxidized cholesterol that is catalyzed by Cyp27A1, and is also abundant in atherosclerotic plaques [10]. 27-hydroxycholesterol has been linked with hypercholesterolemia and metastasis of breast cancer [11, 12]. 27-hydroxycholesterol stimulates cell proliferation of breast cancer, prostate cancer and lung cancer via estrogen receptor [13–15], and enhances breast cancer metastasis via LXR [11]. We previously demonstrated that 27-hydroxycholesterol enhanced osteoclastogenesis in LAC microenvironment, but it is unclear whether 27-hydroxycholesterol is involved in LAC metastasis, and whether 27-hydroxycholesterol links high cholesterol to LAC metastasis.

In the present study, we provided evidences that cholesterol promoted cell invasion of LAC in vitro and LAC metastasis in vivo. Cyp27A1 knockdown not only inhibited the stimulated effect of cholesterol on cell proliferation and invasion. Cyp27A1 deficiency significantly restrained high cholesterol diet-induced LAC metastasis in vivo. These results suggested that 27-hydroxycholesterol was required for LAC metastasis mediated by high cholesterol diet. Mechanism investigation demonstrated that 27-hydroxycholesterol induced the phosphorylation of AKT and NFκB, and promoted the expression of peptidylprolyl isomerase B (PPIB). PPIB inhibition reduced 27-hydroxycholesterol-induced the expression of snail and vimentin. These results indicated that 27-hydroxycholesterol linked high cholesterol to LAC metastasis by elevating PPIB expression.

## Results

### 27-hydroxycholesterol promoted the proliferation and invasion of lung adenocarcinoma cells

**Fig. 1:**
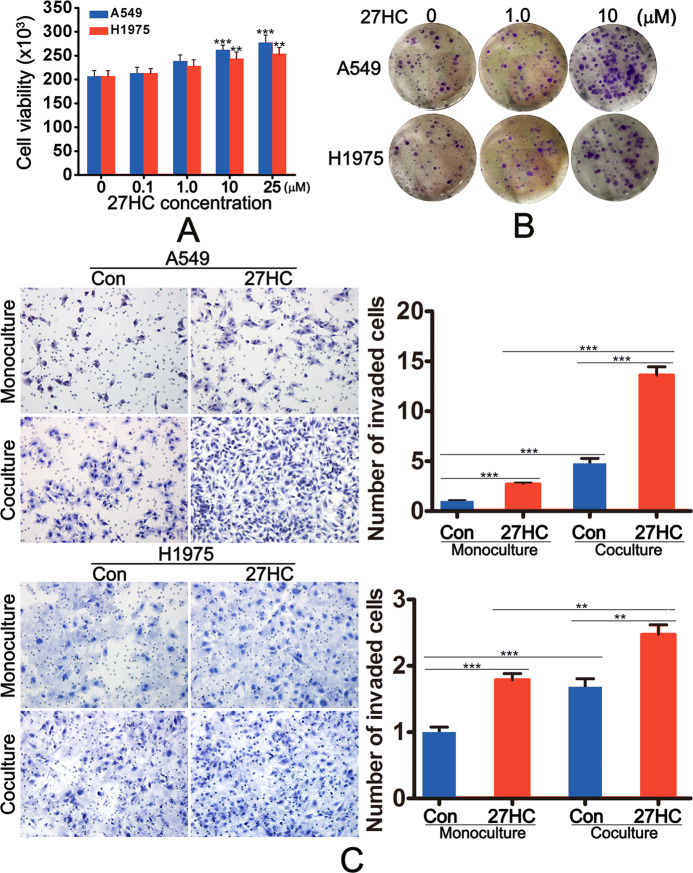
27-HC promoted lung adenocarcinoma proliferation, colony formation, and invasion. **A** A549 and H1975 cells were exposed to different concentration of 27-HC for 24 h. The cell viability was determined using CCK-8 assay. **B** A549 and H1975 cells were treated with 0, 1.0, or 10 μM 27-HC for 14 days, respectively. Colony formation was detected using crystal violet staining. **C** A549 and H1975 cells were treated with 1 μM 27-HC (monoculture system) or cocultured with THP1-derived macrophages treated with 1 μM 27-HC for 24 h. Invasive cells was stained by crystal violet. *P < 0.05; **P < 0.01; ***P < 0.001.

We first tested the effects of 27-hydroxycholesterol (27-HC) on the proliferation and invasion of LAC cells. The CCK-8 assay demonstrated that 27-HC promoted the proliferation of LAC at a dose-dependent manner (Fig. 1A). 10 μM of 27-HC significantly increased cell viability and clone formation (Fig. 1A, B). The scratch assay confirmed that 1 μM of 27-HC accelerated the cell invasion, especially in the coculture system with THP-1-derived macrophage or monocyte-derived macrophage (Figs. 1C, 1s).

### 27-HC was required for cholesterol-induced proliferation and invasion of LAC

**Fig. 2:**
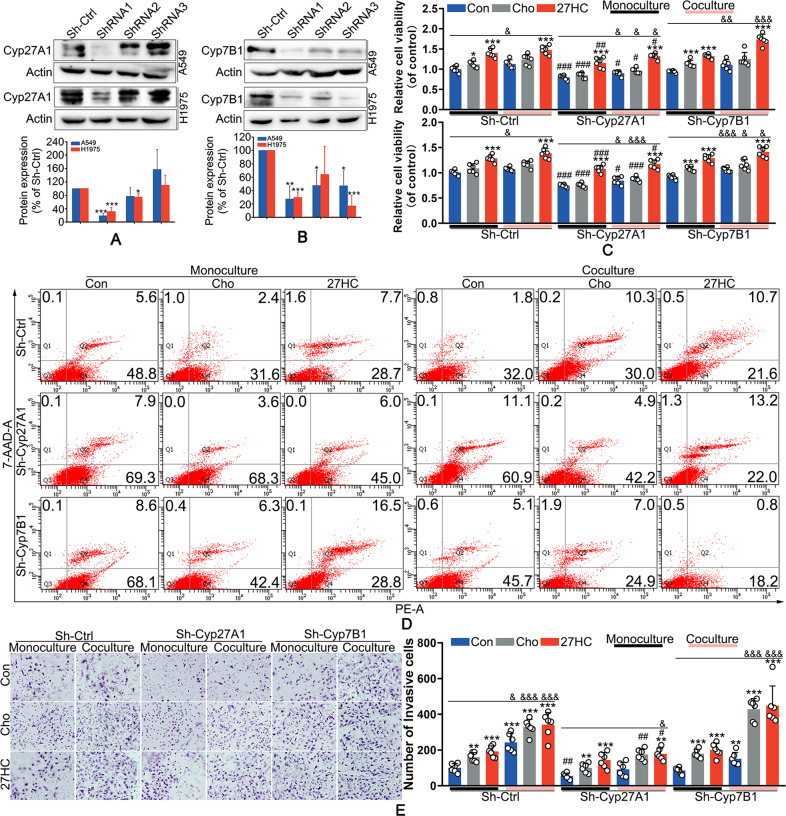
Cyp27A1 knockdown inhibited and Cyp7B1 promoted cholesterol-induced lung adenocarcinoma cells proliferation and invasion. **A**, **B** A549 and H1975 cells were transfected with lentivirus carrying shRNAs targeting Cyp27A1 or Cyp7B1. The expression of Cyp27A1 and Cyp7B1 were measured by western blot. **C** After transfected with shRNAs against Cyp27A1 or Cyp7B1, cells were treated with 1 μM 27-HC or 0.8 mg/ml cholesterol in the monoculture or coculture system with THP1-derived macrophages for 72 h. CCK-8 assay was performed to determine the cell viability. **D** Cells were cultured in monoculture system or coculture system exposed to 1 μM 27-HC or 0.8 mg/ml cholesterol for 72 h. Flow cytometer analysis was performed to determine the cell necrosis and apoptosis. **E** Cells were cultured in monoculture or coculture system exposed to 1 μM 27-HC or 0.8 mg/ml cholesterol for 24 h. Transwell assay were performed to determine cell invasion. *P < 0.05 vs. Con group; **P < 0.01 vs. Con group; ***P < 0.001 vs. Con group; ^#^*P* < 0.05 vs. Sh-Ctrl group; ^##^*P* < 0.01 vs. Sh-Ctrl group; ^###^*P* < 0.001 vs. Sh-Ctrl group; ^&^*P* < 0.05 vs. the corresponding group in the monoculture system; ^&&^*P* < 0.01 vs. the corresponding group in the monoculture system; ^&&&^*P* < 0.001 vs. the corresponding group in the monoculture system.

**Fig. 3:**
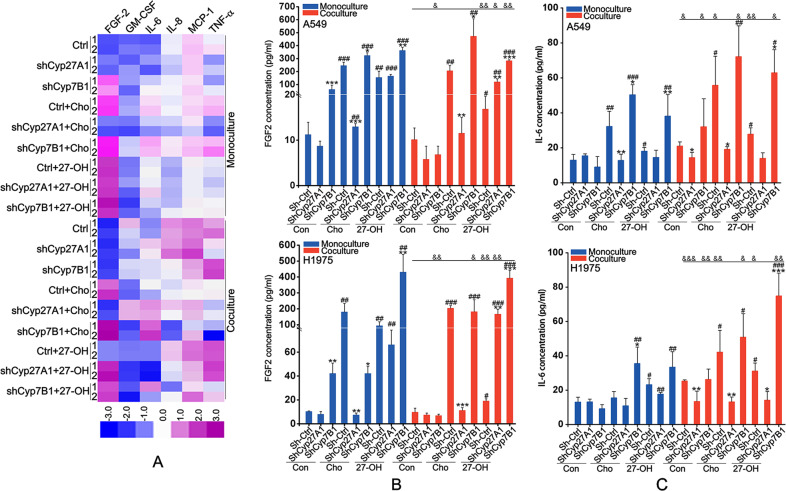
27-HC was essential for cholesterol-induced the release of FGF-2 and IL-6. Cells were cultured in the monoculture or coculture system exposed to 1 μM 27-HC or 0.8 mg/ml cholesterol for 72 h. The supernatants were collected for cytokines analysis using Milliplex Map Multiple cytokines detection kit (**A**) by Luminex 200 and ELISA assay (**B**, **C**). *P < 0.05 vs. Con group; **P < 0.01 vs. Con group; ***P < 0.001 vs. Con group; ^#^*P* < 0.05 vs. Sh-Ctrl group; ^##^*P* < 0.01 vs. Sh-Ctrl group; ^###^*P* < 0.001 vs. Sh-Ctrl group; ^&^*P* < 0.05 vs. the corresponding group in the monoculture system; ^&&^*P* < 0.01 vs. the corresponding group in the monoculture system; ^&&&^*P* < 0.001 vs. the corresponding group in the monoculture system.

Since 27-HC was a metabolite of cholesterol catalyzed by Cyp27A1, knockdown of Cyp27A1 was performed to determine the role of 27-HC in cholesterol-mediated LAC proliferation and invasion. The results showed that among three shRNAs against Cyp27A1, the interference efficiency of shRNA1 was highest (Fig. 2A), which was used in the subsequent experiments. Cyp7B1 is a metabolic enzyme of 27-HC, and Cyp7B1 knockdown leads to 27-HC accumulation. The interference efficiency of shRNA1 against Cyp7B1 was highest among three shRNAs (Fig. 2B), which was used in the subsequent experiments. Cyp27A1 knockdown significantly inhibited cholesterol-induced LAC growth in both monoculture and coculture systems (Figs. 2C, 2s-A). Whatever in the monoculture system or coculture system, Cyp7B1 knockdown notably reduced cell viability, which was rescued by cholesterol or 27-HC treatment (Figs. 2C, 2s-A). Flow cytometry analysis showed that, after 120 h culture, both cholesterol and 27-HC delayed cell apoptosis in monoculture and coculture systems (Fig. 2D). Knockdown of Cyp27A1 or Cyp7B1 promoted cell apoptosis, which was restrained by 27-HC treatment in both monoculture and coculture system (Fig. 2D). Cholesterol mimicked the effect of 27-HC on cells with Cyp27A1 knockdown, but did not significantly alter the apoptosis of cells with Cyp7B1 knockdown (Fig. 2D). These results suggested that 27-HC might be involved in cholesterol-mediated cell proliferation. Moreover, cholesterol promoted LAC invasion in both monoculture and coculture system, which was restrained by Cyp27A1 knockdown and was accelerated by Cyp7B1 knockdown (Figs. 2E, 2s-B). 27-HC mimicked the effect of cholesterol. Further investigation demonstrated that Cyp27A1 knockdown notably reduced 27-HC levels despite in the presence of cholesterol, but did not significantly affect 27-HC levels after 27-HC supplementation. By contrast, Cyp7B1 knockdown increased 27-HC levels, especially after cholesterol and 27-HC stimulation (Fig. 3s). These results suggested that 27-HC might be required for cholesterol-induced proliferation and invasion of LAC.

### 27-HC was required for cholesterol-induced the release of FGF-2 and IL-6

Considering more remarkable effects of cholesterol and 27-HC in coculture system, we further examined the effects of cholesterol and 27-HC on cytokines release using Milliplex Map Multiple cytokines detection kit. The results showed that both cholesterol and 27-HC induced the release of FGF-2 and IL-6, especially in the coculture system (Fig. 3A, Table S1). Cyp27A1 knockdown significantly blocked the effect of cholesterol, but did not affect the effect of 27-HC (Fig. 3A). Cyp7B1 knockdown notably enhanced the effects of cholesterol and 27-HC (Fig. 3A). These results were verified by ELISA assay (Fig. 3B, C), suggesting that 27-HC might be key for cholesterol-induced the release of FGF-2 and IL-6.

### 27-HC was key for cholesterol-induced PPIB expression

**Fig. 4:**
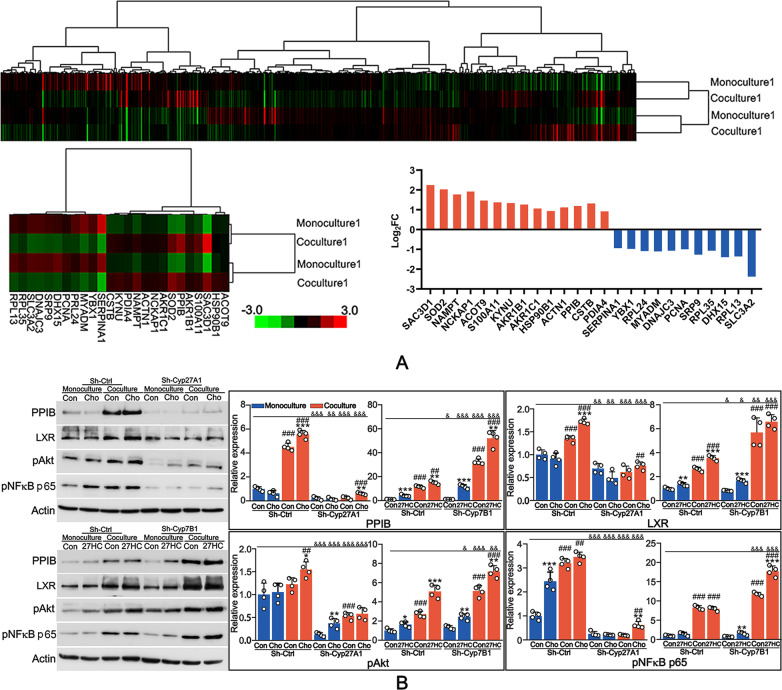
27-HC was key for cholesterol-induced PPIB expression. **A** Cells were cultured in the monoculture system or coculture system exposed to 1 μM 27-HC for 72 h. iTRAQ analysis was performed to determine the differential genes expression. **B** A549 Cells with Cyp27A1 or Cyp7B1 knockdown were cultured in the monoculture system or coculture system exposed to 1 μM 27-HC or 0.8 mg/ml cholesterol for 72 h. Western blot assay was performed to analyze the expression of LXR and PPIB as well as the phosphorylation of AKT and NFκB. *P < 0.05 vs. Con group; **P < 0.01 vs. Con group; ***P < 0.001 vs. Con group; ^#^*P* < 0.05 vs. Sh-Ctrl group; ^##^*P* < 0.01 vs. Sh-Ctrl group; ^###^*P* < 0.001 vs. Sh-Ctrl group; ^&^*P* < 0.05 vs. the corresponding group in the monoculture system; ^&&^*P* < 0.01 vs. the corresponding group in the monoculture system; ^&&&^*P* < 0.001 vs. the corresponding group in the monoculture system.

We next investigated the regulatory functions of 27-HC and cholesterol in signal transduction. iTRAQ analysis revealed that, among 1684 detected proteins, 657 proteins were differentially expressed between monoculture system and coculture system in the presence of 27-HC (Fig. 4A). Further analysis showed that 14 proteins were upregulated more than 1.5-fold and 11 proteins were downregulated more than 1.5-fold (Fig. 4A, Table S2). In the upregulated genes, PPIB expression was increased in both monoculture and coculture system exposed to 27-HC, for which PPIB was selected for subsequent investigation. 27-HC moderately induced PPIB expression in the monoculture system, which was significantly enhanced in the coculture system (Figs. 4B, 4s). Cyp7B1 knockdown strengthened 27-HC-induced PPIB expression in both monoculture and coculture system (Figs. 4B, 4s). Cholesterol treatment did not significantly alter PPIB expression in the monoculture system, but remarkably elevated PPIB expression in the coculture system (Figs. 4B, 4s). Cyp27A1 knockdown almost blocked cholesterol-induced PPIB expression in both monoculture and coculture system (Figs. 4B, 4s). These results suggested that 27-HC might be required for cholesterol-induced PPIB expression. Moreover, in the coculture system, both cholesterol and 27-HC promoted LXR expression and phosphorylation of AKT and NFκB p65 (pAKT and pNFκB p65), which were attenuated by Cyp27A1 knockdown and enhanced by Cyp7B1 knockdown, respectively (Figs. 4B, 4s).

### 27-HC induced PPIB expression by activating NFκB

**Fig. 5:**
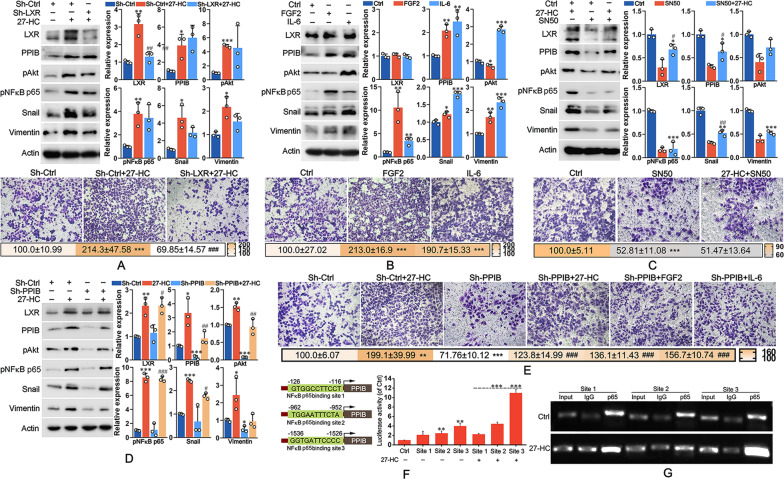
27-HC induced the expression of snail and vimentin by activating LXR and NFκB. **A** A549 cells were transfected with shRNA against LXR, followed by treatment with 1 μM 27-HC for 72 h. Western blot assay was performed to analyze the expression of LXR, PPIB, snail, and vimentin as well as phosphorylation of AKT and NFκB p65. Transwell assay were performed to determine cell invasion. *P < 0.05 vs. Sh-Ctrl group; **P < 0.01 vs. Sh-Ctrl group; ***P < 0.001 vs. Sh-Ctrl group; ^#^*P* < 0.05 vs. 27-HC-treated group; ^##^*P* < 0.01 vs. 27-HC-treated group; ^###^*P* < 0.001 vs. 27-HC-treated group. **B** A549 cells were treated with 10 ng/ml FGF2 or 50 ng/ml IL-6 for 72 h. The levels of the related proteins were determined by western blot analysis. Transwell assay were performed to determine cell invasion. *P < 0.05 vs. Ctrl group; **P < 0.01 vs. Ctrl group; ***P < 0.001 vs. Ctrl group. **C** A549 cells were pretreated with 1 μM SN50 for 6 h, followed by 27-HC stimulation for 72 h. The levels of the related proteins were determined by western blot analysis. Transwell assay were performed to determine cell invasion. *P < 0.05 vs. Ctrl group; **P < 0.01 vs. Ctrl group; ***P < 0.001 vs. Ctrl group; ^#^*P* < 0.05 vs. SN50-treated group; ^##^*P* < 0.01 vs. SN50-treated group, ^###^*P* < 0.001 vs. SN50-treated group. **D** A549 cells were transfected with shRNA against PPIB, and were then treated with 1 μM 27-HC for 72 h. The levels of the related proteins were determined by western blot analysis. **E** After transfected with shRNA against PPIB, cells were treated with 1 μM 27-HC, 10 ng/ml FGF2, or 50 ng/ml IL-6 for 72 h, respectively. Transwell assay were performed to determine cell invasion. *P < 0.05 vs. Sh-Ctrl group; **P < 0.01 vs. Sh-Ctrl group; ***P < 0.001 vs. Sh-Ctrl group; ^#^*P* < 0.05 vs. sh-PPIB-treated group; ^##^*P* < 0.01 vs. sh-PPIB-treated group; ^###^*P* < 0.001 vs. sh-PPIB-treated group. **F** The promoter region of PPIB contains three putative binding sites of NFκB p65. Luciferase reporter assay was performed to determine the activity of PPIB promoter in the presence or absence of 27-HC. *P < 0.05 vs. Sh-Ctrl group; **P < 0.01 vs. Sh-Ctrl group; ***P < 0.001 vs. Sh-Ctrl group; ^#^*P* < 0.05 vs. sh-PPIB-treated group; ^##^*P* < 0.01 vs. sh-PPIB-treated group; ^###^*P* < 0.001 vs. sh-PPIB-treated group. **G** ChIP assay was performed to determine the binding capacity of NFκB p65 on each site of PPIB promoter using specific primers (Site1-F: 5′-CCCGTGCCCCTTGTCCAG-3′, Site1-R: 5′-TCTCCCGCCCACAGGAAG-3′; Site2-F: 5′-CCTGCCTATAATCTGTGCTGT-3′, Site2-R: 5′-CACTTAGTGTTGTCCCAATCT-3′; Site3-F: 5′-GACAGGGTGGAGTGCAGTTTC-3′, Site3-R: 5′-CTTGAGTCCAGGAGTTTGAGC-3′). *P < 0.05; **P < 0.01; ***P < 0.001.

We further explored the molecular mechanism of 27-HC-incuded PPIB expression. As shown in Figs. 5A, 5s, 27-HC induced the expression of LXR, PPIB, Snail and vimentin, and activated AKT and NFκB p65. LXR knockdown partly reduced 27-HC-induced expression of Snail and vimentin, but did not significantly affect the expression of PPIB, pAKT and pNFκB p65, suggesting that 27-HC-induced LAC invasion depended partly on LXR activation. Transwell analysis showed that LXR knockdown indeed reduced 27-HC-induced invasion (Figs. 5A, 5s-A). FGF2 stimulated the expression of PPIB and pNFκB p65, moderately elevated the expression of Snail and vimentin, but did not alter the expression of LXR and pAKT (Figs. 5B, 5s-B). IL-6 exposure elevated the expression of pAKT, pNFκB p65, PPIB, Snail and vimentin, but did not influence the expression of LXR (Figs. 5B, 5s-B). These results suggested that 27-HC-triggered release of FGF2 and IL-6 might accelerate LAC invasion by increasing the expression of pAKT, pNFκB p65, PPIB, Snail, and vimentin. Transwell analysis showed that both FGF2 and IL-6 treatments promoted LAC invasion (Figs. 5B, 5s-B). To determine the role of NFκB p65 in 27-HC-mediated signal transduction, SN50 was used to inhibit the activation of NFκB p65. The results showed that SN50 pretreatment simultaneously reduced the expression of LXR, PPIB, pAKT, pNFκB p65, Snail, and vimentin, despite the presence of 27-HC (Figs. 5C, 5s-C), suggesting an essential role of NFκB p65 in 27-HC-mediated signal transduction. Moreover, SN50 pretreatment inhibited 27-HC-induced LAC invasion (Figs. 5C, 5s-C). Figures 5D, 5s-D illustrated that PPIB knockdown suppressed 27-HC-induced expression of pAKT, Snail and vimentin, but had no notable effects on the expression of LXR and pNFκB p65. Meanwhile, PPIB knockdown reduced LAC invasion, despite in the presence of 27-HC, FGF2, or IL-6 (Figs. 5E, 5s-E). ChiP assay and luciferase reporter assay indicated that NFκB p65 positively regulated the transcription of PPIB (Fig. 5F), which was enhanced by 27-HC treatment. These results suggested that 27-HC might induce PPIB expression to accelerate LAC invasion by activating NFκB.

### High cholesterol diet promoted LAC metastasis in vivo

**Fig. 6:**
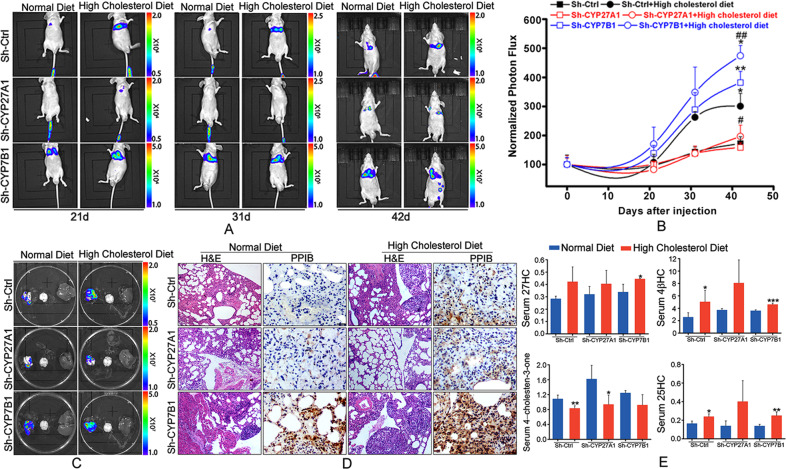
Cyp27A1 knockdown inhibited high cholesterol diet-induced lung adenocarcinoma metastasis and PPIB expression in vivo. **A**, **B** BALB/c nude mice were intravenously inoculated A549 cells (Sh-Ctrl), A549 carrying shRNA against Cyp27A1 (Sh-CYP27A1), or A549 carrying shRNA against Cyp7B1 (Sh-CYP7B1), and feed with normal diet or high cholesterol diet for indicated time. Tumor growth and metastasis were detected using in vivo imaging system. **C** Six weeks after inoculation, mice were sacrificed. The tissues containing lung, brain and liver were obtained and detected using in vivo imaging system. **D** PPIB expression in the lung tissues were tested by immunohistochemical analysis. **E** The levels of 27-HC, 4β-hydroxycholesterol (4βHC), 4-cholesten-3-one, 25-hydroxycholesterol (25-HC) in serum were measured using LC–MS method.

We finally determined the effect of high cholesterol diet on LAC metastasis in vivo. The results showed that high cholesterol diet significantly increased LAC metastasis in vivo compared to normal diet, which was attenuated by Cyp27A1 knockdown and enhanced by Cyp7B1 knockdown (Figs. 6A, B, 6s-A). High cholesterol diet mainly promoted colonization of tumor cells in lung tissue, which was restrained by Cyp27A1 knockdown and strengthened by Cyp7B1 knockdown (Figs. 6C, 6s-B), consistent with Hematoxylin and Eosin (HE) staining (Fig. 6D). In addition, high cholesterol diet elevated PPIB expression, which was inhibited by Cyp27A1 knockdown and elevated by Cyp7B1 knockdown (Fig. 6D). The serum levels of oxycholesterols were determined using LC–MS method. The results showed that high cholesterol diet elevated serum 27-HC level in Cyp7B1 knockdown group (Fig. 6E, Table S3). Furthermore, high cholesterol diet increased the levels of serum 4β-hydroxycholesterol (4βHC) and 25-hydroxycholesterol (25-HC) in control and Cyp7B1 knockdown groups (Fig. 6E). Conversely, high cholesterol diet reduced serum 4-cholesten-3-one level in control and Cyp27A1 knockdown groups (Fig. 6E). These results suggested that high cholesterol diet might accelerate LAC metastasis in vivo by regulating the release of 27-HC, 4βHC, 25-HC, and 4-cholesten-3-one.

### Cyp27A1 deficiency reduced high cholesterol diet-induced LAC metastasis in vivo

**Fig. 7:**
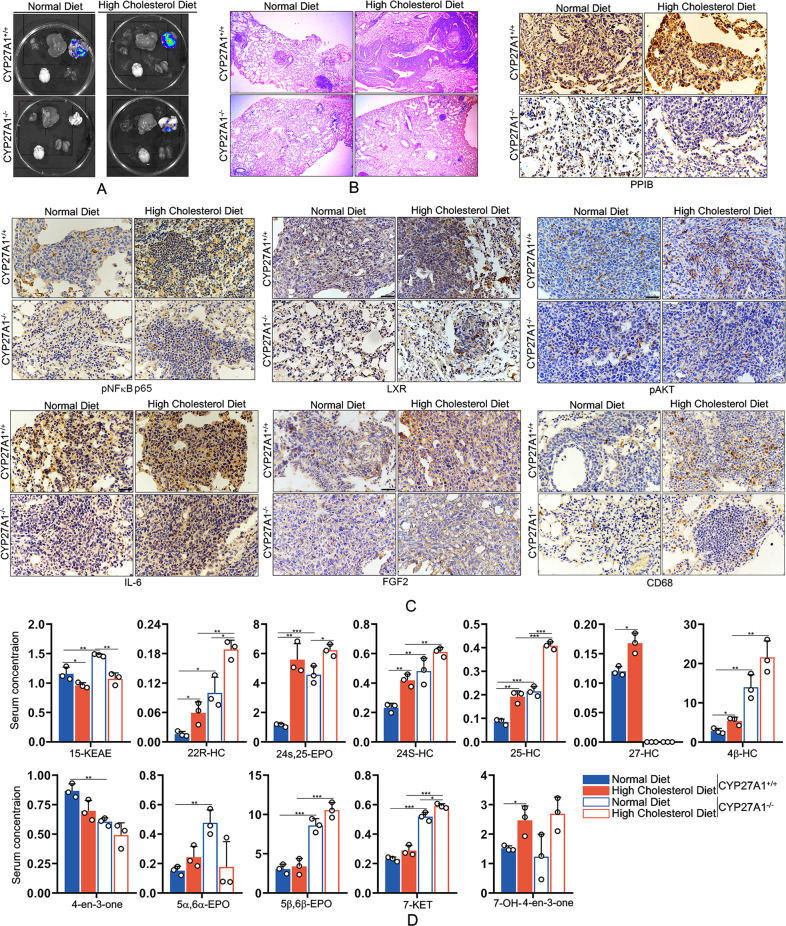
Cyp27A1 deficiency reduced high cholesterol diet-induced LAC metastasis in vivo. Cyp27A1-deficient mice were intravenously inoculated LLC-Luc cells, and feed with normal diet or high cholesterol diet for indicated time. Ten weeks after inoculation, tissues containing lung, brain, liver, and kidney were separated. **A** Fluorescence distributions in tissues were detected using in vivo imaging system. **B** Nodules in tissues were visualized by HE staining. **C** The expression of PPIB, pNFκB p65, LXR, pAKT, IL-6, FGF2, and CD68 were determined by immunohistochemical analysis. **D** The serum levels of oxycholesterols were measured using LC–MS method.

Considering that elevated serum 27-HC might contribute to LAC metastasis, we created Cyp27A1-deficient mice (Cyp27A1^−/−^). After intravenous injection of LAC via tail vein, Cyp27A^+/+^ and Cyp27A^−/−^ mice were divided into two group, and fed with normal diet and high cholesterol diet, respectively. The results showed that high cholesterol diet increased lung metastasis of LAC, which was significantly blocked by Cyp27A1 deficiency (Fig. 7A), consistent with the results from HE staining (Fig. 7B). Immunohistochemistry analysis revealed that high cholesterol diet increased expression of PPIB, LXR, IL-6 and FGF2, and phosphorylation of NFκB p65 and AKT, which simultaneously inhibited by Cyp27A1 deficiency (Fig. 7C). In addition, high cholesterol diet-induced macrophage infiltration (CD68 positive cells) in tumor microenvironment, which was reduced by Cyp27A1 deficiency (Fig. 7C). Serological analysis showed that high cholesterol diet elevated serum 27-HC level, which was remarkably blocked by Cyp27A1 deficiency (Fig. 7D, Table S4). Moreover, high cholesterol diet stimulated the secretion of 22R-hydroxycholesterol (22R-HC), 24s, 25-epoxycholesterol (24s, 25-EPO), 25-HC, 4βHC and 7-hydroxy-4-cholesten-3-one (7-OH-4-en-3-one) (Fig. 7D, Table S4). These results suggested that Cyp27A1 deficiency might inhibit high cholesterol diet-induced LAC metastasis by regulating the secretion of several oxycholesterols, especially 27-HC.

## Discussion

Cholesterol is an important component of lipid raft on the mammalian cell membrane, and mediates series of intracellular signal transduction. Recently, cholesterol metabolism has attracted great attentions in the field of tumors. Increasing evidences reveal that cholesterol and its metabolites accelerate tumor progression, such as breast, prostate and colon cancers [14, 16, 17]. However, the role of cholesterol metabolism in LAC is controversial and remains largely unknown. A recent meta-analysis reveals that dietary cholesterol intake is positively associated with lung cancer risk in the case-control studies with a total of 6894 lung cancer cases and 29,736 controls, which could not be evidenced in the cohort studies with 241,920 participants and 1769 lung cancer cases [18]. Another meta-analysis about cohort studies shows that serum total cholesterol is inversely associated with lung caner risk [19]. Thus, it is necessary to explore the molecular mechanism of cholesterol and its metabolites in lung cancer. In this study, we demonstrated that high cholesterol diet accelerated in vivo metastasis of LAC, which was inhibited by Cyp27A1 knockout. Considering Cyp27A1 is a key enzyme that catalyzes the hydroxylation of cholesterol to 27-hydroxycholesterol (27-HC), we speculated that 27-HC played an important role in cholesterol-mediated LAC metastasis. 27-HC treatment promoted the proliferation and invasion of LAC, especially in the coculture system with macrophage, similar to the effect of cholesterol stimulation. Cyp27A1 knockdown inhibited cholesterol-induced cell proliferation and invasion. Cyp7B1 is a metabolic enzyme of 27-HC, and its knockdown promoted cholesterol-mediated cell proliferation and invasion. These results suggested an essential role of 27-HC in cholesterol-induced proliferation and invasion. 27-HC is an abundant cholesterol metabolite that links hypercholesterolemia and breast cancer metastasis by activating LXR [11]. In LAC cells, LXR expression was elevated in the coculture system with THP1-derived macrophage, which might enhance the function of 27-HC.

Mechanism investigation showed that both 27-HC and cholesterol induced the phosphorylation of AKT and NFκB as well as PPIB expression, especially in the coculture system. Intriguingly, LXR knockdown did not affect the expression of these molecules except EMT related factors snail and vimentin. These results indicated that 27-HC acted on cell migratory abilities not only through activating LXR, but also via activating AKT and NFκB. There are little reports about the function of 27-HC in NFκB pathway. Valeria et al. [20] reveal that 27-HC enhances the effect of NFκB activation on HSV-1 infection. Our results confirmed that pretreatment with SN50, a specific inhibitor of NFκB, simultaneously inhibited the expression of snail, vimentin and PPIB, suggesting that NFκB activation was main reason for 27-HC-induced expression of snail, vimentin and PPIB. Luciferase assay and ChIP assay revealed that NFκB mediated the transcription of PPIB. PPIB knockdown inhibited the expression of snail and vimentin. PPIB, also named cyclophilin B, has been implicated in chemoresistance and radioresistance of tumors [21, 22], but is largely undiscovered in tumor metastasis. Our results evidenced that PPIB played a key role in cholesterol-induced and 27-HC-induced signal transduction as well as cells proliferation and invasion. In addition, 27-HC stimulated the secretion of FGF2 and IL-6, which also contributed to activate NFκB and increased the expression of PPIB, snail and vimentin. It has been testified that FGF2 and IL-6 induce EMT in LAC [23, 24]. These results indicated that, on the one hand, 27-HC promoted the expression of snail and vimentin by activating LXR, facilitating the proliferation and invasion of LAC; on the other hand, 27-HC induced the expression of PPIB, snail and vimentin by activating NFκB and increasing secretion of FGF2 and IL-6, accelerating cells invasion.

## Conclusions

**Fig. 8:**
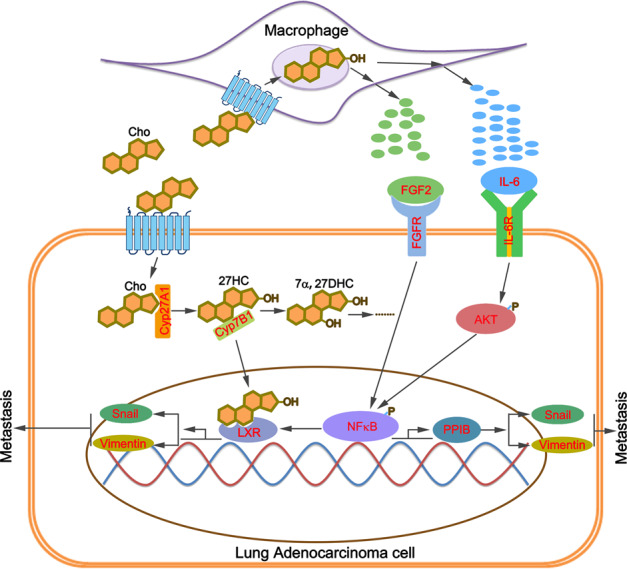
The schematic diagram. The possible functional mechanism of 27-HC in high cholesterol-induced lung adenocarcinoma metastasis.

In this study, we evidenced that cholesterol promoted cell proliferation and invasion of LAC in vitro and LAC metastasis in vivo, which was inhibited by Cyp27A1 knockdown or deficiency. These results indicated that 27-hydroxycholesterol was key for LAC invasion and metastasis induced by cholesterol. Mechanism investigation demonstrated that 27-hydroxycholesterol induced the expression of snail and vimentin by activating NFκB/PPIB axis and LXR as well as promoting the secretion of FGF2 and IL-6 (Fig. 8), which accounted for cholesterol-induced LAC invasion and metastasis.

## Materials and methods

### Materials

The antibodies were used as follows: anti-Cyp27A1, anti-Cyp7B1, anti-Akt (mAb, #4685), anti-phospho-Akt (mAb, #4060) and anti-phospho-NF-κB p65 (mAb, #3033) were purchased from Cell Signaling Technology (Beverly, MA); anti-PPIB (pAb, ab16045), anti-LXR (pAb, ab24362), anti-FGF2 (mAb, ab92337) and anti-IL-6 (pAb, ab6672) were purchased from Abcam (Cambridge, MA); anti-beta-actin (mAb, #66009-1-Ig) was purchased from Proteintech (Proteintech group Inc, Rosement, US). RIPA lysis buffer was purchased from Cell Signaling Technology (Beverly, MA). Water-soluble cholesterol was purchased from Sigma Chemical Co. (USA). 27-hydroxycholesterol (27-HC) was obtained from Yuanye Biological Techonology (Shanghai, China). Dulbecco’s modified Eagle’s medium (DMEM) and fetal bovine serum (FBS) were purchased from Gibco Life Technologies (Grand Island, NY, USA).

### Cell culture

The LAC cells A549 and H1975 were purchased from American Type Culture Collection (Manassas, VA), and were cultured in DMEM containing 10% FBS, 100 IU/ml penicillin, and 100 mg/ml streptomycin at 37 °C in a humidified atmosphere of 5% CO_2_. THP-1 cells were cultured in DMEM with 10% FBS, penicillin/streptomycin, and 100 ng Phorbol 12-Myristate 13-Acetate (PMA) for 48 h. To exclude the effect of PMA, THP1-derived macrophages were cultured in DMEM with 10% FBS for another 24 h.

### Monocyte-derived macrophage differentiation

The peripheral blood mononuclear cells (PBMCs) were isolated from peripheral blood of healthy donors by centrifugation in Ficoll. PBMCs were cultured at a density of 5 × 10^6^ cells/well in 12-well plates containing serum free DMEM medium for 2 h. After removing non-adherent cells, the adherent cells were washed using sterile PBS and replaced with fresh DMEM with 20% FBS. The cells were cultured for 7 days with medium exchange every 3 days.

### Cell treatment

27-hydroxycholesterol (27-HC) was dissolved in ethanol before use. Water-soluble cholesterol (Cho) was diluted with DMEM medium before use. The solvents were used as control groups, respectively. In the monoculture system, A549 and H1975 cells were cultured overnight, starved for 4 h, and were then supplemented with 1% FBS and different dose of Cho or 27-HC. In the coculture system, THP1-derived macrophages or monocyte-derived macrophage were placed in the bottom chamber containing DMEM with 10% FBS, indicated dose of Cho or 27-HC. A549 or H1975 cells were seeded into the upper chambers containing free-serum DMEM.

### Cell viability assay

Cells were seeded in 96-well plates at a density of 1 × 10^4^ cells/well and cultured overnight. After starved for 4 h, cells were exposed to 1 μM 27-HC or 0.8 mg/ml Cho for different time, followed by incubation with 10 μl CCK-8 solution for 2 h at 37 °C. The absorbance was detected at 450 nm using a Multiskan GO spectrophotometer. All experiments were performed in triplicates.

### Clonogenic assay

A549 and H1975 cells were seeded at a density of 800 cells/well in 6-well plates and cultured with indicated concentration of 27-HC for 14 days, exchanging media every 2 days. Subsequently, the colonies stained using 1% crystal violet (Sigma) were captured by microscope.

### Invasion assay

A549 and H1975 cells were seeded at a density of 1 × 10^5^ cells/well into the upper chambers coated with Matrigel Matrix (BD, Biosciences, USA). THP1-derived macrophages were seeded at a density of 2 × 10^5^ cells/well into the bottom chamber containing DMEM with 10% FBS, indicated dose of Cho or 27-HC. After 24 h of culture, the cells on the lower surface of the membranes were counted using the crystal violet staining (Sigma).

### Cytokine assay

After indicated treatments, the cell supernatants were collected for analysis of cytokines. The concentrations of FGF2, GM-CSF, IL-17α, IL-1β, IL-6, IL-8, MCP-1, MIP-1α, and TNF-α were measured by Milliplex Map Multiple cytokines detection kit using Luminex 200. The differential cytokines were verified by ELISA assay.

### iTRAQ analysis

The treated cells were lysed in RIPA lysis buffer containing 0.1% SDS. After quantification, 200 μg of protein was denatured and digested by trypsin overnight at 37 °C. The generated peptides were labeled by iTRAQ reagents, and were then mixed to be identified and quantified using LC–MS/MS.

### Gene transfection

A549 H1975 cells were seeded at a density of 1 × 10^3^ cells/well in 96-well plates and cultured overnight. The cells were then replaced in fresh media and transfected with lentivirus carrying shRNA targeting Cyp27A1 (5′-CATTGTCCTGGTTCCCAAT-3′), Cyp7B1 (5′-ACCTCACCAGAGAACAATT-3′) or PPIB (5′-GGTGGAGAGCACCAAGACA-3′).

### Western blot

Total protein was extracted using RIPA lysis buffer (Thermo), and was then subjected to SDS-PAGE. The protein was subsequently transferred onto PVDF membranes. The membrane was blocked with 5% fat-free milk and incubated with the primary antibodies and the corresponding secondary antibodies. The protein signals were visualized using the ECL detection system and quantified by Scion Image 4.03 software.

### Immunohistochemistry

Tumor sections were embedded in paraffin, followed by treatment with xylene and ethanol. After antigen retrieval by microwave treatment, the samples were incubated with 3% hydrogen peroxide to remove endogenous peroxidase. Subsequently, the samples were blocked using 10% goat serum, and were then incubated with primary antibodies and the corresponding secondary antibodies.

### Luciferase reporter assay

The sequence containing NFκB p65 binding site 1, 2, or 3 was cloned into pGL3-Basic vector to construct pGL3-site1, pGL3-site2, and pGL3-site3, respectively. The A549 cells were transfected with pGL3-site1, pGL3-site2, or pGL3-site3 plasmid using Lipofectamine 3000 (Roche), followed by treatment with 27-HC. After incubation for 24 h, cellular lysates were obtained to determine dual luciferase activity according to the manufacturer’s instructions.

### Flow cytometer analysis

Cells necrosis and apoptosis were determined using flow cytometer analysis. Cells were collected using trypsin without EDTA and phenol red, and were then incubated with PE-conjugated annexin-V reagent and 7-AAD, followed by flow cytometer analysis.

### In vivo LAC model

Six-week-old male BALB/c nude mice were purchased from Beijing Vital River Animal Center (Beijing, China) and maintained under specific pathogen-free conditions in the animal facility. All procedures were approved by Laboratory Animal Ethics Committee of Shandong Provincial Hospital Affiliated to Shandong First Medical University. Each mouse was intravenously inoculated 2 × 10^6^ A549 cells, A549 carrying shRNA against Cyp27A1, or A549 carrying shRNA against CYP7B1 in 100 μl sterile PBS via tail vein, followed by feed with normal diet or high cholesterol diet (97.5% normal diet, 2% cholesterol and 0.5% sodium cholate), respectively. Three weeks after inoculation, tumor growth and metastasis were detected using in vivo imaging system (IVIS Lumina XRMS Series III, PerkinElmer). Six weeks after inoculation, mice were sacrificed and tissues containing lung, brain and liver were fixed in 4% formaldehyde and embedded in paraffin for HE and immunohistochemistry staining analysis.

Cyp27A1^+/−^ mice were generated by GemPharmatech Co., Ltd (Nanjing, China). Cyp27A1^−/−^ mice were identified by genotyping to be used for In vivo tumorigenesis experiment. Lewis-derived LAC cells (LLC) were tranfected with lentiviral vector carrying luciferase to create LLC-Luc cells. Six-week-old Cyp27A1^−/−^ or control mice, half male and half female, were intravenously inoculated 2 × 10^6^ LLC-Luc cells in 100 μl sterile PBS via tail vein, and subsequently fed with normal diet or high cholesterol diet (97.5% normal diet, 2% cholesterol and 0.5% sodium cholate), respectively. Ten weeks after inoculation, mice were sacrificed and tissues containing lung, brain, liver and kidney were fixed in 4% formaldehyde and embedded in paraffin for HE and immunohistochemistry staining analysis.

### Statistical analysis

GraphPad Prism 8.0 software was used to carry out statistical analysis. All data are shown as mean ± SD from at least three independent experiments. The Student’s *t* test was used to assess the difference between two groups. One-way ANOVA was used to evaluate the difference among multiple groups. *P* < 0.05 was statistically significant.

## Supplementary information

Table S1

Table S2

Table S3

Table S4

Supplementary Figure

## Data Availability

All data generated or analyzed during this study are included in this published article (and its Supplementary Information files).
