# Supplementary material for: 27-hydroxycholesterol linked high cholesterol diet to lung adenocarcinoma metastasis
Source: Oncogene. 2022 Apr 4;41(19):2685–95. doi: 10.1038/s41388-022-02285-y (PMC9076535; doi:10.1038/s41388-022-02285-y)
Supplement: Supplementary file 1 — Table S1 [file 41388_2022_2285_MOESM1_ESM.pdf]

Table S1 Effect of cholesterol (Cho) and 27-hydroxycholesterol (27HC) on secretion of FGF2 and IL6 in monoculture or coculture system

	FGF2				IL6			
Sample	MFI(i)	pg/ml(i	MFI	pg/ml	MFI(i)	pg/ml(i	MFI	pg/ml
A549-1	14	10.5	14	10.5	202.5	11.27	202.5	11.27
A549-2	13	8.94	13	8.94	238	13.22	238	13.22
A549+Sh-A1-1	13.5	9.76	13.5	9.76	242	13.44	242	13.44
A549+Sh-A1-2	13	8.94	12.5	8.76	157	8.69	157	8.69
A549+Sh-B1-1	100	103.9	100	103.94	370	20.17	370	20.17
A549+Sh-B1-2	78	84.28	78	84.28	306.5	16.88	298.8	16.47
A549+Cho-1	269.5	229.7	269.5	229.67	1148	57.17	1149	57.17
A549+Cho-2	270	230	274.5	233.03	1262	62.37	1222	60.54
A549+Sh-A1+Cho-1	13.5	9.72	13.25	9.33	474.5	25.43	474.5	25.43
A549+Sh-A1+Cho-2	14	10.5	14	10.5	449.5	24.19	449.5	24.19
A549+Sh-B1+Cho-1	303	252	294.5	246.4	1262	62.37	1222	60.54
A549+Sh-B1+Cho-2	382	303.4	382	303.41	1148	57.17	1149	57.17
A549+27HC-1	175	163.3	175	163.27	937	47.44	937	47.44
A549+27HC-2	184	169.9	184	169.89	1262	62.37	1222	60.54
A549+Sh-A1+27HC-1	120	120.7	120	120.71	1422	69.69	1422	69.69
A549+Sh-A1+27HC-2	126	125.6	126	125.57	1106	55.22	1106	55.22
A549+Sh-B1+27HC-1	382	303.4	382	303.41	1306	64.38	1306	64.38
A549+Sh-B1+27HC-2	303	252	294.5	246.4	2154	103.3	1983	95.35
A549+M-1	12	7.38	12	7.38	306.5	16.88	298.8	16.47
A549+M-2	14	10.5	14	10.5	330.5	18.14	330.5	18.14
A549+Sh-A1+M-1	11	5.82	11	5.82	202.5	11.27	202.5	11.27
A549+Sh-A1+M-2	10.5	5.04	10.5	5.04	334	18.32	334	18.32
A549+Sh-B1+M-1	12	7.38	12	7.38	20915	2438	20915	2438
A549+Sh-B1+M-2	14	10.5	13.5	9.72	207.5	11.55	207.5	11.55
A549+M+Cho-1	286	240.7	286	240.74	1055	52.88	1055	52.88
A549+M+Cho-2	175	163.3	175	163.27	1516	73.96	1516	73.96
A549+Sh-A1+M+Cho-1	12	7.38	12	7.38	776	39.93	776	39.93
A549+Sh-A1+M+Cho-2	16	13.56	16	13.56	911	46.23	911	46.23
A549+Sh-B1+M+Cho-1	726.5	519.1	748.8	532.98	3444	171.5	3447	171.5
A549+Sh-B1+M+Cho-2	564	418	564	417.97	4744	233.2	4744	233.2
A549+M+27HC-1	17	15.07	17	15.07	276	15.27	271.5	15.03
A549+M+27HC-2	18	16.56	17.5	15.82	238	13.22	238	13.22
A549+Sh-A1+M+27HC-	126	125.6	126	125.57	84	4.36	84	4.36
A549+Sh-A1+M+27HC-	78	84.28	78	84.28	157	8.69	157	8.69
A549+Sh-B1+M+27HC-	382	303.4	382	303.41	3686	213.2	3686	213.2
A549+Sh-B1+M+27HC-	290	243.4	290	243.41	1262	62.37	1222	60.54

M, represented THP1-derived macrophage
