# Supplementary material for: 27-hydroxycholesterol linked high cholesterol diet to lung adenocarcinoma metastasis
Source: Oncogene. 2022 Apr 4;41(19):2685–95. doi: 10.1038/s41388-022-02285-y (PMC9076535; doi:10.1038/s41388-022-02285-y)
Supplement: Supplementary file 2 — Table S2 [file 41388_2022_2285_MOESM2_ESM.pdf]

Table S2 1.5 fold differentiated genes between monoculture system and coculture system in the presence of 27-HC

Name	monoculture1	monoculture2	colculture1	colculture2
SAC3D1	1.202263951	0.946237087	5.754398823	4.325138092
SOD2	0.809095919	0.751622915	3.076097012	3.250873089
NAMPT	0.602559626	0.591561615	1.836537957	2.208004951
NCKAP1	0.270395786	0.270395786	1.018591046	1
ACOT9	3.16E-01	0.383707315	0.801678121	1.116863012
S100A11	1.018591046	0.88715601	2.728977919	2.167704105
KYNU	0.591561615	0.772680581	1.644371986	1.770109057
AKR1B1	0.794328213	0.724435985	1.940886021	1.659587026
AKR1C1	0.847227395	0.758577585	1.659587026	1.67494297
HSP90B1	0.895364821	0.794328213	1.458814025	1.737800956
ACTN1	0.642687678	0.608134985	1.355188966	1.330453992
PPIB	2.070141077	1.981663942	4.920394897	4.256278038
CSTB	0.597035289	0.794328213	1.737800956	1.706081986
PDIA4	0.990831971	0.912010789	1.753880978	1.819700956
SERPINA1	6.668066978	5.754398823	3.53183198	2.992264986
YBX1	1.58776999	1.247382998	0.801678121	0.642687678
RPL24	1.541700006	1.047129035	0.698232412	0.534564376
MYADM	2.870682001	2.42E+00	1.29419601	1.177524448
DNAJC3	1.923092008	1.923092008	0.928966403	0.912010789
PCNA	8.17E-01	0.963828981	0.4487454	0.452897608
SRP9	1.43E+00	1.541700006	0.625172675	0.613762021
RPL35	0.963828981	1.037528038	0.580764413	0.383707315
DHX15	1.406048059	1.803017974	0.63573	0.591561615
RPL13	1.30E+00	1.499685049	0.660693526	0.432513803
SLC3A2	1.2592	1.318256974	0.21887991	0.280543387
