# Supplementary material for: 27-hydroxycholesterol linked high cholesterol diet to lung adenocarcinoma metastasis
Source: Oncogene. 2022 Apr 4;41(19):2685–95. doi: 10.1038/s41388-022-02285-y (PMC9076535; doi:10.1038/s41388-022-02285-y)
Supplement: Supplementary file 3 — Table S3 [file 41388_2022_2285_MOESM3_ESM.pdf]

Table S3 After mouse was inoculated A549 cells, A549 carrying shRNA against Cyp27A1, or A549 carrying shRNA against CYP7B1 via tail vein, the serum oxycholesterols were determined using LC-MS method.

	Normal diet				High cholesterol diet				Normal diet			
**Compoun**	**Sh-Ctrl1**	**Sh-Ctrl2**	**Sh-Ctrl3**	**Sh-Ctrl4**	**Sh-Ctrl1**	**Sh-Ctrl2**	**Sh-Ctrl3**	**Sh-Ctrl4**	**Sh-27A1-1**	**Sh-27A1-2**	**Sh-27A1-3**	**Sh-27A1-4**
22r-CHO	0.018523	0.024612	0.01969	0.027956	0.025785	0.031228	0.029151	0.045806	0.036719	0.030753	0.021842	0.021241
24s-CHO	0.251515	0.259483	0.266667	0.238667	0.281166	0.277632	0.23066	0.245161	0.291016	0.306276	0.199123	0.21383
27-HCO	0.291288	0.309483	0.258915	0.276	0.299103	0.339912	0.495283	0.550968	0.371094	0.379079	0.259211	0.271631
4b-HCO	3.189189	3.190349	1.963054	1.895408	6.537468	6.754617	3.467492	3.395904	3.414634	3.609023	3.882979	3.955556
5a,6a-Epo	0.135676	0.198928	0.288177	0.375	0.103876	0.12533	0.162848	0.203413	0.324121	0.397561	0.632979	0.675556
5b,6b_Epo	0.835135	1.008043	1.490148	1.663265	0.728682	0.82058	0.897833	0.863481	1.620603	1.909756	2.569149	2.755556
7a-HCO	0.261745	0.294043	0.282278	0.252324	0.231754	0.254408	0.299248	0.352727	0.174065	0.189677	0.221364	0.231377
4-Choleste	1.202685	1.13308	1.027848	0.97344	0.760563	0.783375	0.912782	0.863636	1.238318	1.367742	1.970109	1.897143
7-KET	0.101894	0.115748	0.117668	0.133459	0.097665	0.101931	0.08301	0.083978	0.094	0.105686	0.157563	0.152443
7-OH-4-Ch	0.16553	0.255906	0.277032	0.29812	0.115175	0.164479	0.245631	0.279006	0.100667	0.102341	0.147899	0.152117
1a-hydroxy	0.07803	0.072441	0.083392	0.093233	0.097665	0.106564	0.15534	0.19558	0.118	0.124749	0.161765	0.192508
24s,25_Ep	0.298864	0.252756	0.770318	0.642857	0.224125	0.22471	0.472816	0.392265	0.35	0.319064	0.516807	0.442997
15a-hydrox	0.110204	0.156109	0.238376	0.291905	0.264929	0.260099	0.179703	0.229333	0.180567	0.197009	0.198578	0.216942
6a-hydroxy	0.123265	0.153846	0.172694	0.235238	0.219905	0.241872	0.130693	0.156667	0.189069	0.221795	0.296209	0.321901
25-CHO	0.132245	0.153846	0.172694	0.196667	0.206161	0.27734	0.228713	0.242	0.195547	0.174786	0.093365	0.090909
	High cholesterol diet				Normal diet				High cholesterol diet			
**Compoun**	**Sh-27A1-1**	**Sh-27A1-2**	**Sh-27A1-3**	**Sh-27A1-4**	**Sh-7B1-1**	**Sh-7B1-2**	**Sh-7B1-3**	**Sh-7B1-4**	**Sh-7B1-1**	**Sh-7B1-2**	**Sh-7B1-3**	**Sh-7B1-4**
22r-CHO	0.031088	0.033252	0.031374	0.035922	0.027444	0.033807	0.026018	0.04029	0.026712	0.032831	0.020402	0.021583
24s-CHO	0.334728	0.369919	0.269466	0.28	0.25	0.267045	0.304977	0.301932	0.328767	0.312785	0.273092	0.260618
27-HCO	0.493724	0.504065	0.310305	0.30902	0.271486	0.310425	0.353394	0.41875	0.451142	0.446119	0.437778	0.442029
4b-HCO	10.9893	11.59686	4.924623	4.756098	3.458824	3.530806	3.608815	3.814433	4.914773	4.664804	4.37931	4.501608
5a,6a-Epo	0.097861	0.115183	0.108537	0.138847	0.098621	0.111576	0.30854	0.343643	0.380682	0.430168	0.694118	1.00237
5b,6b_Epo	0.617647	0.657068	0.604878	0.764411	0.434483	0.4791	1.37741	1.237113	1.46875	1.659218	3.647059	4.218009
7a-HCO	0.236413	0.188571	0.257692	0.248775	0.213208	0.256993	0.173134	0.219585	0.325798	0.244709	0.202153	0.247045
4-Choleste	0.741313	0.689616	1.187179	1.117647	1.326415	1.188811	1.265957	1.193853	0.962687	1.115079	1.086603	0.508902
7-KET	0.100373	0.095668	0.079004	0.072449	0.051711	0.058373	0.081301	0.095238	0.091941	0.104511	0.166438	0.176125
7-OH-4-Ch	0.082836	0.088448	0.228114	0.264286	0.105348	0.139234	0.121951	0.141126	0.483516	0.511278	0.359589	0.380623
1a-hydroxy	0.075	0.064621	0.036299	0.10034	0.117112	0.115789	0.171138	0.267532	0.112454	0.121805	0.115411	0.1609
24s,25_Ep	1.600746	1.418773	0.33452	0.259184	0.366845	0.286124	1.130081	0.939394	0.758242	0.699248	0.558219	0.525952
15a-hydrox	0.50431	0.522321	0.223188	0.285776	0.353143	0.480519	0.385859	0.396791	0.269863	0.26682	0.304603	0.327948
6a-hydroxy	0.206897	0.220089	0.113406	0.146121	0.121143	0.157792	0.223737	0.204278	0.216895	0.241014	0.379079	0.475983
25-CHO	0.551724	0.633929	0.187681	0.228017	0.142009	0.124424	0.123013	0.160699	0.215429	0.222727	0.272727	0.293583

Sh-A1, represented knockdown of Cyp27A1; Sh-B1, represented knockdown of Cyp7B1.
