# Supplementary material for: 27-hydroxycholesterol linked high cholesterol diet to lung adenocarcinoma metastasis
Source: Oncogene. 2022 Apr 4;41(19):2685–95. doi: 10.1038/s41388-022-02285-y (PMC9076535; doi:10.1038/s41388-022-02285-y)
Supplement: Supplementary file 4 — Table S4 [file 41388_2022_2285_MOESM4_ESM.pdf]

Table S4 The effect of Cyp27A1 deficiency on serum oxycholesterols in the presence of normal diet or high cholesterol diet.

	$Cyp27A1^{+/+}$						$Cyp27A1^{-/-}$					
	Normal diet			High cholesterol diet			Normal diet			High cholesterol diet		
**Oxysterol Metabolites**	A1	A2	A3	B1	B2	B3	C1	C2	C3	D1	D2	D3
**15-ketocholestane**	1.284	1.070	1.119	1.012	0.922	0.949	1.477	1.451	1.493	1.102	1.154	0.962
**22(R)-Hydroxycholester**	0.014	0.022	0.012	0.079	0.064	0.035	0.088	0.136	0.076	0.204	0.167	0.194
**24(S),25-Epoxycholeste**	1.208	1.184	1.012	4.864	6.837	5.063	4.011	4.507	5.189	6.213	5.828	6.646
**24(S)-Hydroxycholester**	0.253	0.198	0.247	0.460	0.422	0.376	0.566	0.490	0.390	0.637	0.619	0.577
**25-Hydroxycholesterol**	0.071	0.094	0.091	0.205	0.163	0.208	0.237	0.212	0.195	0.425	0.393	0.409
**27-Hydroxycholesterol**	0.129	0.114	0.117	0.185	0.168	0.151	0.000	0.000	0.000	0.000	0.000	0.000
**4b-hydroxycholesterol**	3.560	2.293	2.584	5.857	4.269	6.033	17.459	11.204	13.304	26.133	17.934	20.806
**4-cholesten-3-one**	0.855	0.932	0.815	0.679	0.620	0.794	0.569	0.635	0.611	0.529	0.572	0.375
**5a,6a-Epoxycholesterol**	0.156	0.179	0.120	0.326	0.184	0.219	0.458	0.572	0.400	0.376	0.077	0.075
**5b,6b-Epoxycholesterol**	2.439	3.664	3.080	4.364	3.446	2.343	9.456	8.638	7.713	10.507	11.560	9.603
**7-Oxocholesterol**	0.243	0.234	0.213	0.272	0.329	0.262	0.548	0.519	0.489	0.588	0.607	0.578
**7-OH-4-Cholesten-3-one**	1.617	1.465	1.464	1.932	2.896	2.577	1.257	1.987	0.462	2.101	3.225	2.746
**cholestane-triol**	0.583	0.637	0.790	0.967	0.664	0.632	0.550	0.614	0.774	0.828	0.853	0.718
