# Supplementary material for: 27-hydroxycholesterol linked high cholesterol diet to lung adenocarcinoma metastasis
Source: Oncogene. 2022 Apr 4;41(19):2685–95. doi: 10.1038/s41388-022-02285-y (PMC9076535; doi:10.1038/s41388-022-02285-y)
Supplement: Supplementary file 5 — Supplementary Figure [file 41388_2022_2285_MOESM5_ESM.pdf]

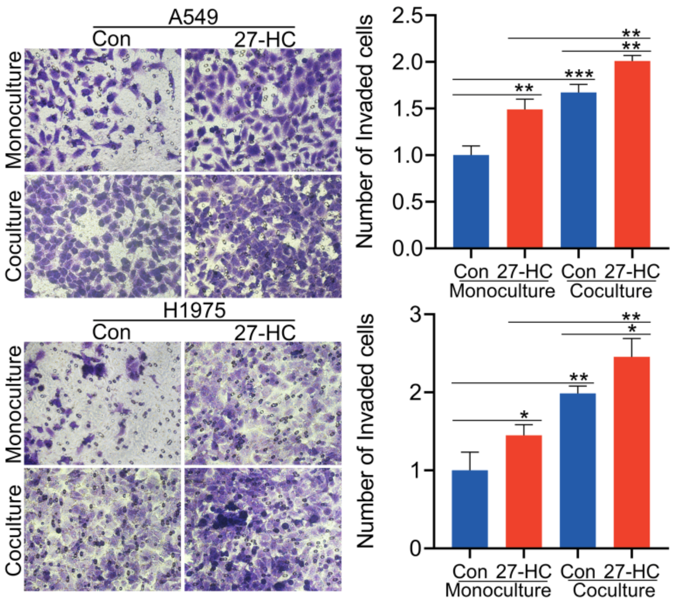

Fig.1s A549 and H1975 cells were treated with 1 μM 27-HC (monoculture system) or cocultured with monocyte-derived macrophages treated with 1 μM 27-HC for 24 h. Invasive cells was stained by crystal violet. *P < 0.05; ** P<0.01; *** P<0.001.

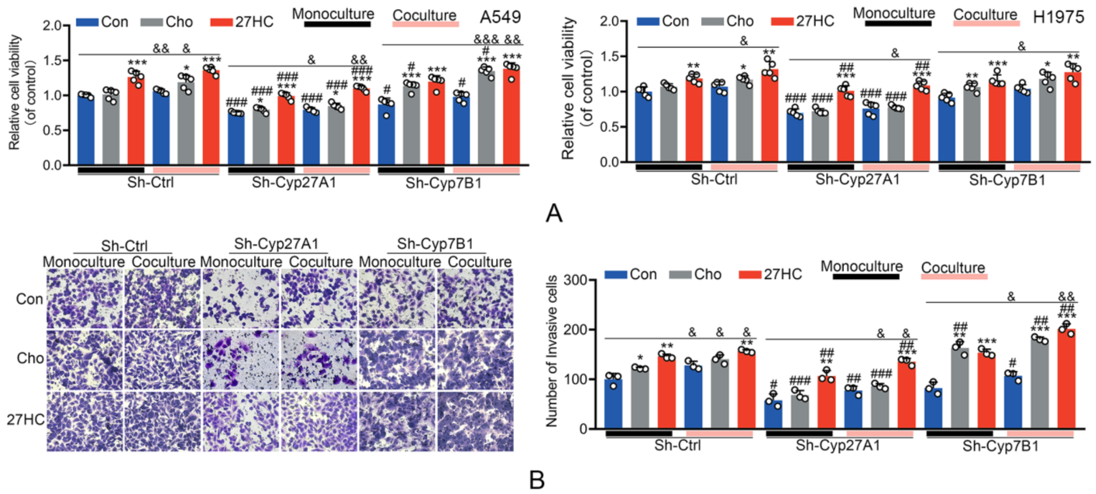

Fig.2s A. Lung adenocarcinoma cells with Cyp27A1 or Cyp7B1 knockdown were treated with 1 μM 27-HC or 0.8 mg/ml cholesterol in the monoculture or coculture system with monocyte-derived macrophages for 72 h. Cell viability was determined by CCK8 assay. B. A549 cells with Cyp27A1 or Cyp7B1 knockdown were treated with 1 μM 27-HC or 0.8 mg/ml cholesterol in the monoculture or coculture system with monocyte-derived macrophages for 24 h. Transwell assay were performed to determine cell invasion. *P < 0.05 vs Con group; ** P<0.01 vs Con group; *** P<0.001 vs Con group; #P < 0.05 vs Sh-Ctrl group; ## P<0.01 vs Sh-Ctrl group; ###P<0.001 vs Sh-Ctrl group; &P < 0.05 vs the corresponding group in the monoculture system; && P<0.01 vs the corresponding group in the monoculture system; &&&P<0.001 vs the corresponding group in the monoculture system.

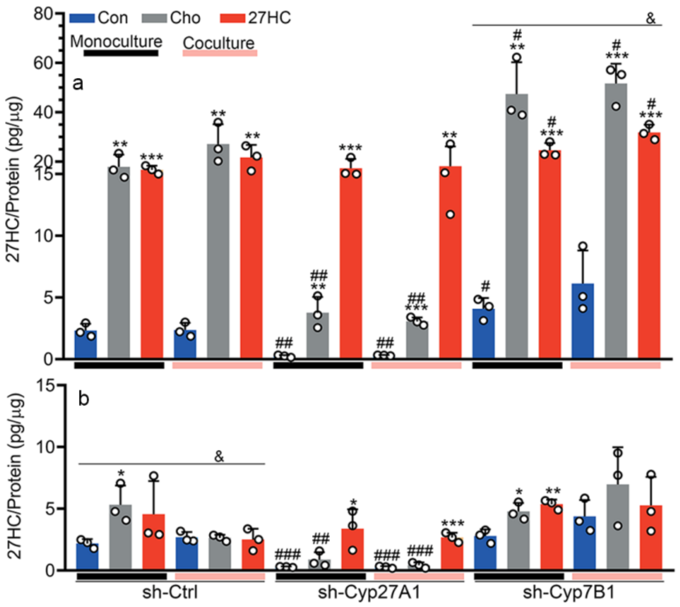

Fig.3s Lung adenocarcinoma cells with Cyp27A1 or Cyp7B1 knockdown were treated with 1 μM 27-HC or 0.8 mg/ml cholesterol in the monoculture or coculture system with THP1-derived macrophages for 2 h (a) or 72 h (b). Intracellular 27-HC levels were determined using LC-MS method.

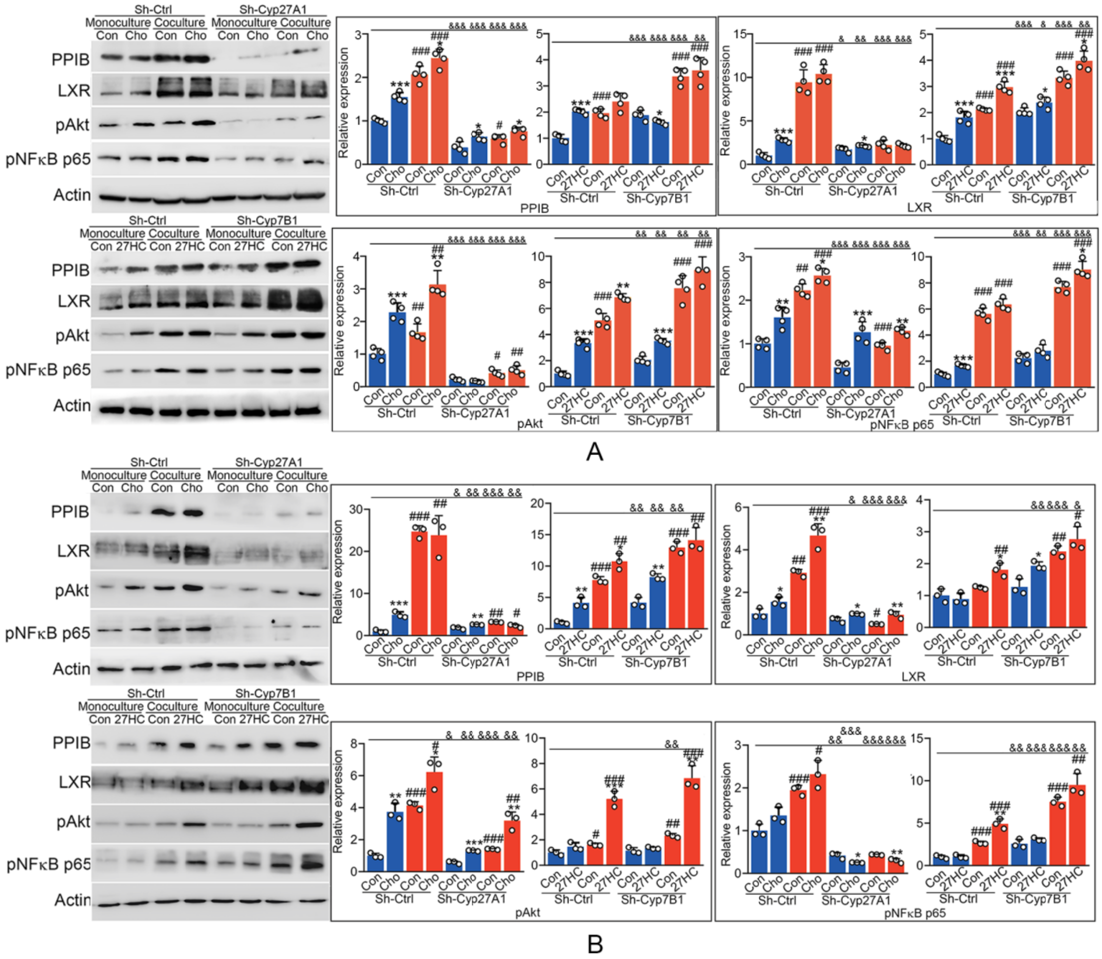

Fig.4s A. H1975 Cells with Cyp27A1 or Cyp7B1 knockdown were cultured in the monoculture system or coculture system with THP1-derived macrophages exposed to 1 μM 27-HC or 0.8 mg/ml cholesterol for 72 h. Western blot assay was performed to analyze the expression of LXR and PPIB as well as the phosphorylation of AKT and NFκB. B. A549 Cells with Cyp27A1 or Cyp7B1 knockdown were cultured in the monoculture system or coculture system with monocyte-derived macrophages exposed to 1 μM 27-HC or 0.8 mg/ml cholesterol for 72 h. The relative protein expression was determined by western blot analysis. *P < 0.05 vs Con group; ** P<0.01 vs Con group; *** P<0.001 vs Con group; #P < 0.05 vs Sh-Ctrl group; ## P<0.01 vs Sh-Ctrl group; ###P<0.001 vs Sh-Ctrl group; &P < 0.05 vs the corresponding group in the monoculture system; && P<0.01 vs the corresponding group in the monoculture system; &&&P<0.001 vs the corresponding group in the monoculture system.

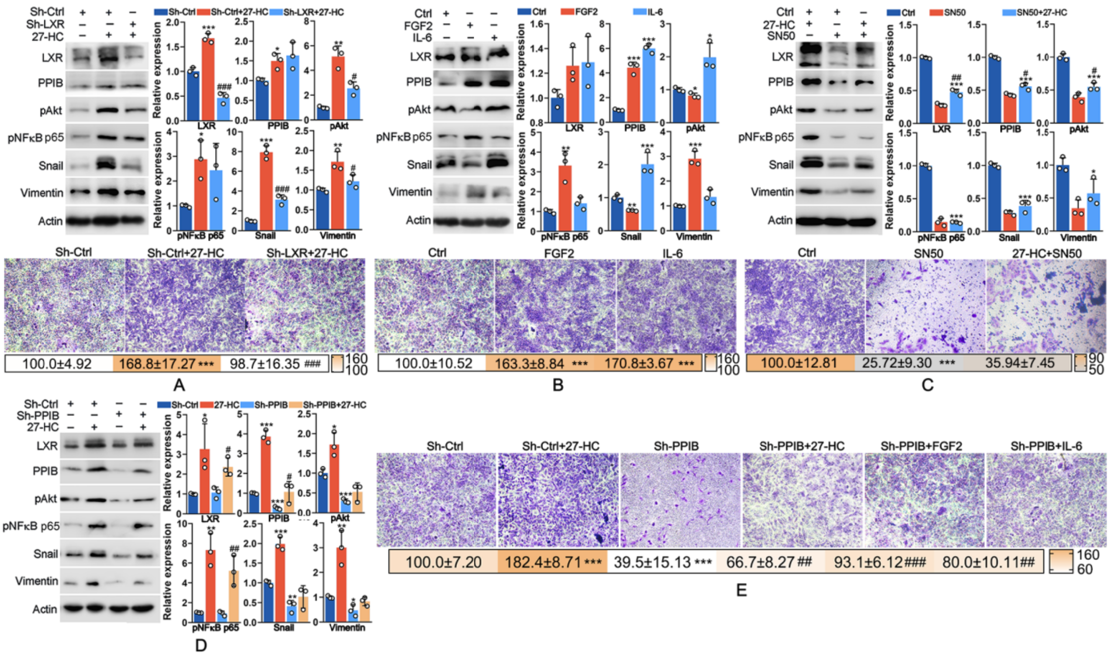

Fig.5s A. H1975 cells were transfected with shRNA against LXR, followed by treatment with 1 μM 27-HC for 72 h. Western blot assay was performed to analyze the expression of LXR, PPIB, snail and vimentin as well as phosphorylation of AKT and NFκB p65. Transwell assay were performed to determine cell invasion. *P < 0.05 vs Sh-Ctrl group; ** P<0.01 vs Sh-Ctrl group; *** P<0.001 vs Sh-Ctrl group; # P < 0.05 vs 27-HC-treated group; ## P < 0.01 vs 27-HC-treated group; ### P < 0.001 vs 27-HC-treated group. B. H1975 cells were treated with 10 ng/ml FGF2 or 50 ng/ml IL-6 for 72 h. The related protein expression was determined by western blot analysis. Transwell assay were performed to determine cell invasion. *P < 0.05 vs Ctrl group; ** P<0.01 vs Ctrl group; *** P<0.001 vs Ctrl group. C. H1975 cells were pretreated with 1 μM SN50 for 6 h, followed by 27-HC stimulation for 72 h. The related protein expression was determined by western blot analysis. Transwell assay were performed to determine cell invasion. *P < 0.05 vs Ctrl group; ** P<0.01 vs Ctrl group; *** P<0.001 vs Ctrl group; # P < 0.05 vs SN50-treated group; ## P < 0.01 vs SN50-treated group. D. H1975 cells were transfected with shRNA against PPIB, and were then treated with 1 μM 27-HC for 72 h. The related protein expression was determined by western blot analysis. E. After transfected with shRNA against PPIB, cells were treated with 1 μM 27-HC, 10 ng/ml FGF2 or 50 ng/ml IL-6 for 72 h, respectively. Transwell assay were performed to determine cell invasion. *P < 0.05 vs

Sh-Ctrl group; ** $P<0.01$ vs Sh-Ctrl group; *** $P<0.001$ vs Sh-Ctrl group; # $P < 0.05$ vs sh-PPIB-treated group; ## $P < 0.01$ vs sh-PPIB-treated group; ### $P < 0.001$ vs sh-PPIB-treated group.

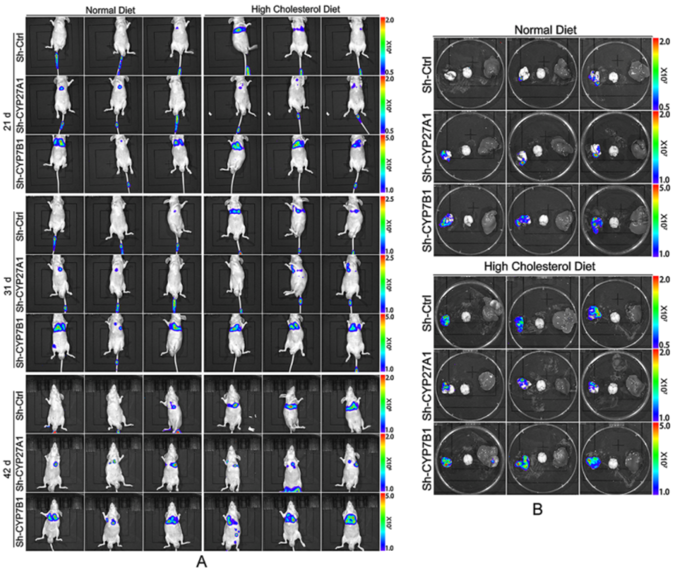

Fig.6s A. BALB/c nude mice were intravenously inoculated A549 cells (Sh-Ctrl), A549 carrying shRNA against Cyp27A1 (Sh-CYP27A1), or A549 carrying shRNA against Cyp7B1 (Sh-CYP7B1), and feed with normal diet or high cholesterol diet for indicated time. Tumor growth and metastasis were detected using in vivo imaging system. B. Six weeks after inoculation, mice were sacrificed. The tissues containing lung, brain and liver were obtained and detected using in vivo imaging system.
